# Left Ventricular Outflow Tract Obstruction Due to Cardiac Hamartoma

**DOI:** 10.7759/cureus.62721

**Published:** 2024-06-19

**Authors:** Witina Techasatian, Andrew Pham, Yoshito Nishimura, Dipanjan Banerjee

**Affiliations:** 1 Medicine, John A. Burns School of Medicine, University of Hawaii, Honolulu, USA

**Keywords:** hamartoma, cardio-oncology, hamartoma of mature cardiac myocytes, left ventricular outlet obstruction, cardiac hamartoma

## Abstract

Hamartoma of mature cardiac myocytes (HMCM) is a rare, benign cardiac tumor. We report a case of a 19-year-old female with an atypical presentation, including significant weight loss and abnormal electrocardiogram. A transthoracic echocardiogram (TTE) revealed a mass causing left ventricular outflow tract (LVOT) obstruction, confirmed by cardiac magnetic resonance (CMR) imaging showing a 5 x 3 cm mass contiguous with the right ventricular free wall and exhibiting heterogeneous, diffuse late gadolinium enhancement. The patient subsequently underwent sternotomy for surgical biopsy and septal myectomy, with histology of the mass being consistent with HMCM. The patient remained asymptomatic during her 6-month follow-up.

## Introduction

Cardiac hamartoma is a benign cardiac tumor that stems from the overgrowth of cardiac cells mixed with various other tissues. Hamartoma of mature cardiac myocytes (HMCM) is one of the rare tumors characterized by localized, disorganized, and hypertrophied mature cardiac myocytes with proliferated blood vessels [1]. While patients with HMCM generally remain undiagnosed or asymptomatic, they may have serious cardiac complications. Here, we report a case of HMCM in a young female who initially presented with nonspecific symptoms and was found to have left ventricular outflow tract (LVOT) obstruction.

## Case presentation

**Figure 1:**
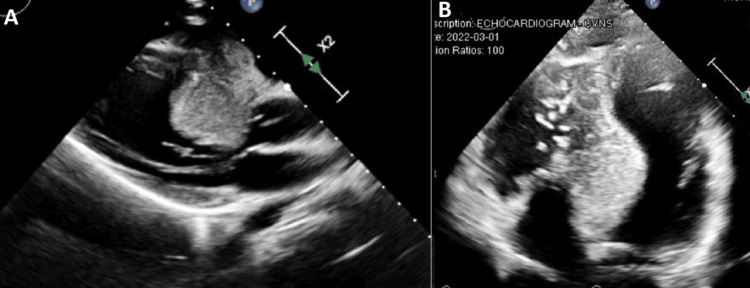
TTE (A) Parasternal long axis view and (B) apical four-chamber view. A large mass in the interventricular septum measuring 5 x 5 x 3 cm in diameter. There appears to be partial obliteration of the interventricular septum. TTE, transthoracic echocardiogram

A 19-year-old female with no significant past medical history presented to the clinic with unintentional weight loss, 13.6 kg over six months (23% of usual body weight). An electrocardiogram was ordered prior to inpatient treatment for presumed anorexia, which showed nonspecific T wave inversions. The patient otherwise felt well and denied any abnormal cardiac symptoms, including shortness of breath, chest pain, palpitation, or syncope. Given a short, systolic murmur noted at the left sternal border on physical examination, a transthoracic echocardiogram (TTE) was ordered to exclude structural cardiac abnormalities. TTE revealed a large mass in the interventricular septum with partial obliteration of the interventricular septum (Figure 1).

**Figure 2:**
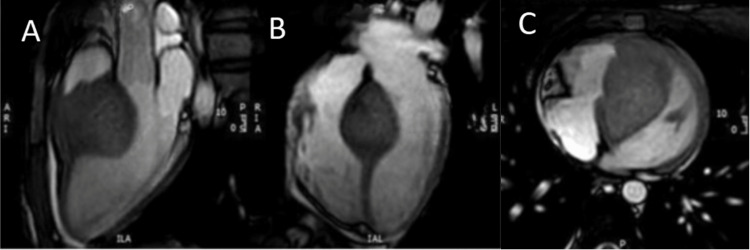
CMR imaging (A and B) SSFP of apical four-chamber and apical three-chamber images in sequence. (C) Mid axial SSFP image showing continuity with the free wall. Large interventricular mass involving the basal-mid septum measuring 5 x 3 cm. CMR, cardiac magnetic resonance; SSFP, steady-state free precession

Both visual and color Doppler assessments demonstrated LVOT obstruction caused by the mass. Cardiac magnetic resonance (CMR) imaging was subsequently performed. Subsequently, CMR confirmed a large interventricular mass involving the basal-mid septum and measuring 5 x 3 cm (Figure 2).

It appeared contiguous with the right ventricular free wall with heterogeneous diffuse late gadolinium enhancement. Given concerns for possible cardiac sarcoma based on the CMR imaging, computed tomography angiography was ordered. It revealed a large ventricular septal mass with a relatively well-encapsulated appearance and hypervascularity. The decision was made to proceed with a right ventricular endomyocardial biopsy, which revealed only mild myocyte hypertrophy. A fluorodeoxyglucose (FDG)-positron emission tomography (PET) scan found mild diffuse FDG uptake in the mass. The patient underwent an open sternotomy for surgical biopsy to rule out malignancy. Gross findings showed pink-tan to white-tan tissue fragments. Pathology findings demonstrated myocyte hypertrophy with disarray, intimately associated with increased variably dilated veins and intramyocardial arteries. The immunostaining was positive for CD31 and negative for CAMTA1, which was most consistent with HMCM. Due to the diagnostic challenges, the decision was made to proceed with a two-step surgical approach for definitive treatment. The patient underwent sternotomy for right and left side ventricular debulking and septal myectomy. The tumor and interventricular septum were debulked until the left and right ventricular outflow tracts were wide open. After a six-month follow-up, she remained asymptomatic, was able to exercise, and recently enrolled in a university class.

## Discussion

To our knowledge, this is the first reported case of HMCM complicated by LVOT obstruction. While the epidemiology and characteristics of this tumor are not well described, the left ventricle appears to be the most common location of HMCM [2]. A definitive diagnosis requires tissue pathology. Surgical treatment may be needed for definitive diagnosis and symptom relief. The present case illustrates the clinical challenges of evaluating a newly diagnosed cardiac mass. Given the difficulty of differential diagnosis in cardiac hamartoma and the risks related to open-heart biopsy, intraoperative rapid histopathology is essential in such cases.

Distinguishing HMCM from other cardiac masses can be challenging. Multimodality imaging should be used for initial screening, including echocardiogram, computed tomography, and CMR imaging. The gold standard for diagnosis is a tissue specimen. Histologically, HMCM is composed of hypertrophic, disorganized mature cardiac myocytes with sarcoplasmic vacuolization. Frequently, it is associated with proliferated blood vessels [3]. Given the need to exclude malignancy and the similarity of HMCM to normal cardiac tissue without definitive histopathological findings, HMCM can be considered a diagnosis of exclusion.

The differential diagnosis of HMCM includes benign etiologies, such as rhabdomyoma and hypertrophic cardiomyopathy (HCM), and malignant cardiac tumors. One example of such malignant tumors is angiosarcoma, a highly invasive cancer that can resemble HMCM from its vascular appearance. However, the location of angiosarcoma is more commonly in the right atrium, whereas HMCM reportedly occurs more in the left ventricle. Histology demonstrating endothelial differentiation with the formation of anastomosing channels lined by atypical cells with mitotic activity is the main feature of diagnosed angiosarcoma [1]. Another diagnosis to consider is rhabdomyoma, one of the most common benign cardiac tumors closely resembling HMCM on imaging. However, rhabdomyoma is characterized by rounded myocytes with large vacuoles and intervening strands of myocyte cytoplasm. Moreover, most cases are pediatric, as it is strongly associated with tuberous sclerosis [4]. In addition, HMCM, located in the septal or ventricular area, could mimic HCM as histological features are almost identical without good pathological distinction, causing misdiagnosis in some patients [3,5,6]. A family history of HCM and more diffuse myocardial involvement with septal localization may help to distinguish HCM from HMCM.

The management of cardiac hamartoma consists of close observation of symptoms and surgical intervention if mechanical obstruction or arrhythmia is attributed to the tumor present. Complete surgical excision or partial debulking surgery has been performed successfully with significant improvement of symptoms such as arrhythmia and dyspnea [6]. Following surgery, periodic clinical evaluations and echocardiograms are necessary to monitor for possible recurrence. If the tumor recurs, surgical resection may need to be considered based on the location and symptomatology of the recurrence.

## Conclusions

In patients with mechanical complications, an upfront surgical approach may be preferred to avoid complications from repeated endomyocardial biopsies. The surgical intervention, whether complete or partial resection of the mass, should be considered based on the location of the mass. In a patient with a large septal mass, we recommend intraoperative histopathology to exclude malignancy. Partial resection to reduce LVOT is preferred over more extensive surgery to avoid complications.
